# Associations between musculoskeletal pain and work-related factors among public service sector computer workers in Kaunas County, Lithuania

**DOI:** 10.1186/s12891-016-1281-7

**Published:** 2016-10-07

**Authors:** Gintare Kaliniene, Ruta Ustinaviciene, Lina Skemiene, Vidmantas Vaiciulis, Paulius Vasilavicius

**Affiliations:** Department of Environmental and Occupational Medicine, Public Health Faculty, Lithuanian University of Health Sciences, Tilzes 18, Kaunas, LT-47181 Lithuania

**Keywords:** Musculoskeletal pain, Computer work, Ergonomics, RULA

## Abstract

**Background:**

Information technologies in occupational activities have been developing very rapid. Epidemiological studies have shown that musculoskeletal disorders are widely prevalent among employees working with a computer. The aim of this study was to evaluate the prevalence of musculoskeletal pain in various anatomical areas and its associations with individual, ergonomic, and psychosocial factors among computer workers of the public sector in Kaunas County, Lithuania.

**Methods:**

The investigation consisting of two parts – questionnaire study (Nordic Musculoskeletal Questionnaire and Copenhagen Psychosocial Questionnaire) and direct observation (evaluation of work ergonomics using the Rapid Upper Limb Assessment [RULA]) – was carried out in three randomly selected public sector companies of Kaunas County. The representative study sample comprised 513 public service office workers. The prevalence of musculoskeletal pain in five anatomical areas of the body (shoulders, elbows, wrists/hands, as well as upper and low back) was evaluated.

**Results:**

The prevalence rates of shoulder, elbow, wrist/hand, upper and low back pain were 50.5 %, 20.3 %, 26.3 %, 44.8 %, and 56.1 %, respectively. Individual factors such as gender, age, computer work experience, and body mass index were found as significant for musculoskeletal pain in various musculoskeletal regions. The respondents reporting pain in shoulder, wrist/hand, upper back, and low back areas had a statistically significantly higher mean RULA score. The duration of working with a computer was found as a significant factor for shoulder pain. High quantitative demands were related to musculoskeletal pain in all investigated anatomical areas expect for the low back; weak social support was a significant predictor for complaints in upper and low back areas.

**Conclusion:**

This study confirmed associations between musculoskeletal pain and work ergonomics; therefore, preventive measures at the workplace should be directed to the improvement in ergonomic work environment, education, and workload optimization.

## Background

Musculoskeletal (MS) disorders are the most common work-related health problem in Europe, affecting millions of workers [1]. It is also the largest group of occupational diseases accounting for about one-third and more of all registered occupational diseases in the United States, Scandinavian countries, and Japan [2].

Information technologies have become indispensable in the office environment, which has led to intensified computer use. Many epidemiological studies show that MS complaints are widely prevalent among employees working with a computer [3–10]. Scientific reports indicate that computer users mostly report complaints about pain the neck area [7–11]. Our recent survey on MS complaints in the neck and their associations with work-related factors has shown a very high prevalence (65.7 %) of MS pain in this area during a 12-month period [12]. However, complaints about pain in other anatomical body regions (shoulders, upper extremities, back) are also prevalent among computer users. Significant associations of these complaints with both physical [10, 13–15] and psychosocial [16–19] work environment are noted in studies. Research confirms that the working environment is not the only factor that has an impact on the development of MS disorders – individual characteristics such as gender, age, and body mass index (BMI) are also significant [6, 20–22]. Some studies have reported evidence that MS disorders have a multifactorial origin [3, 23, 24]; however, other systematical reviews have not confirmed psychosocial factors to have a predictive value for MS complaints [25] or have not found even moderate evidence to confirm causative relationship between computer work and diagnosed MS disorders [26]. With the intention to fully consider the predisposing aspects of MS disorders, all factors – individual, physical, and psychosocial – were analyzed in this study.

The aim of this study was to evaluate the prevalence of MS pain in various anatomical areas and its associations with individual, ergonomic, and psychosocial factors among computer workers in the public sector of Kaunas County, Lithuania.

## Methods

This study was part of the cross-sectional epidemiologic study “Ergonomics of computer work and its interface with work environment”, carried out at the Department of Environmental and Occupational Medicine, Lithuanian University of Health Science, in 2010.

Scientific literature reports that frequency of the event of interest – MS disorders – in computer working populations varies from 6.6 % to 70 %. The sample size calculation was based on the frequency with 5 % probability of error and 95 % reliability, and 0.5 relative frequency [27], and this resulted in 384 participants needed to complete the study. Three institutions from the list of 12 public service institutions in Kaunas County were randomly selected to recruit participants. Employees whose work ware directly related to computer use were invited to participate in the study. A total of 570 questionnaires were distributed among employees, and 513 employees agreed to participate in the study and completed the questionnaire properly (response rate, 89.1 %).

The study protocol was approved by Kaunas Regional Ethics Committee for Biomedical Research, Lithuania (Protocol No. BE-2-13). The participation in the study was anonymous and voluntary; written informed consent to participate in the study was obtained from each participant together with the completed questionnaire.

### Questionnaires

**Fig. 1:**
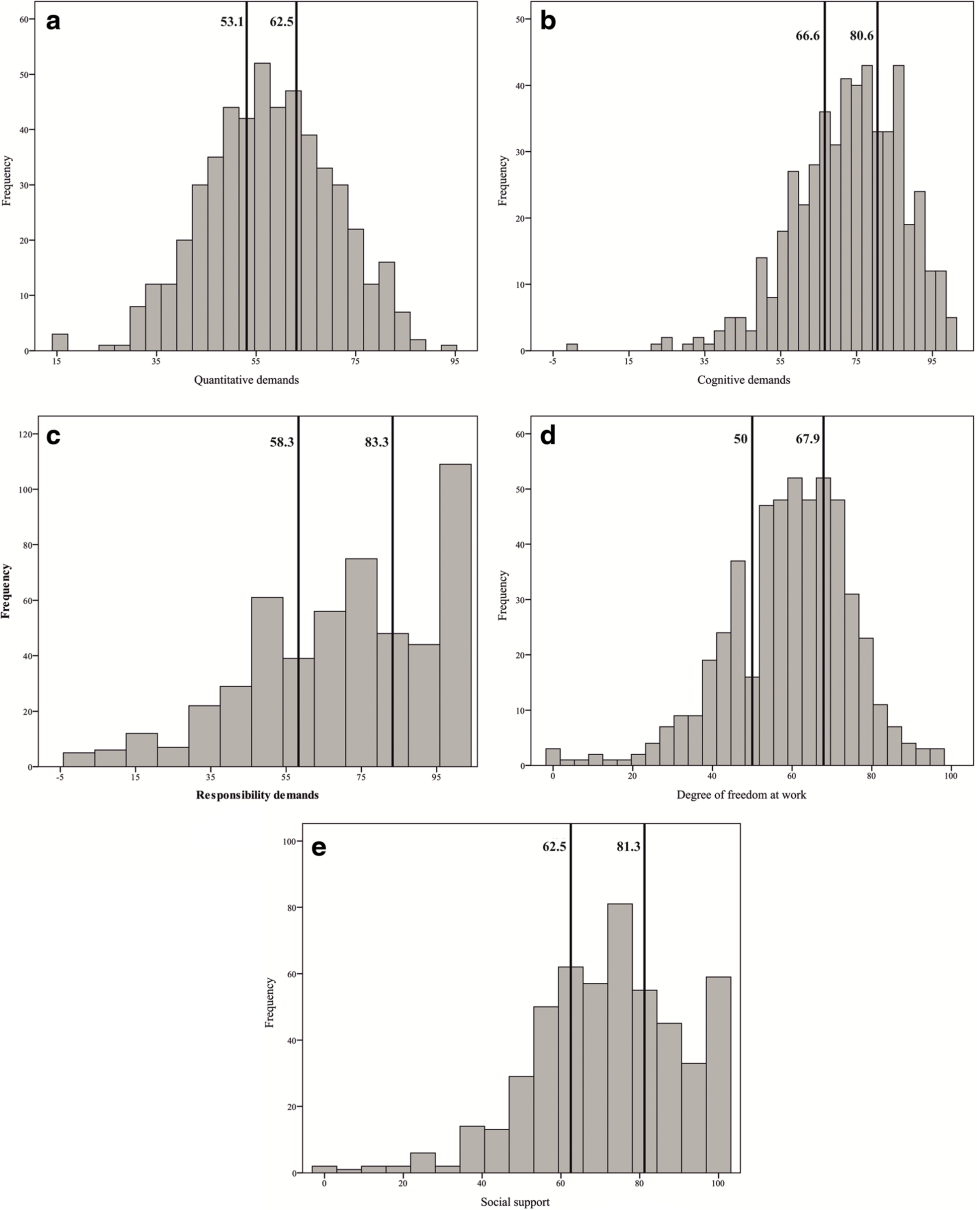
Tertile margins of psychosocial characteristics: **a** Quantitative demands, **b** Cognitive demands, **c** Responsibility demands, **d** Degree of freedom at work, **e** Social support

A three-part questionnaire was used in this study. The first part of the questionnaire included the questions that were designed to gather individual data of the respondents (age, gender, height, weight, and computer work experience). The division into age categories was done for the purpose of more convenient statistical analysis; the participants were divided into four age groups. Computer work experience was categorized into three groups. The second part was intended to assess the 12-month prevalence of MS pain involving five anatomical areas of the body: shoulders, elbows, wrists/hands, as well as upper and low back. For this purpose, the Nordic Musculoskeletal Questionnaire was used [28]. The standardized Copenhagen Psychosocial Questionnaire was employed in order to evaluate the psychosocial work environment [29]. Five scales (quantitative demands, cognitive demands, responsibility demands, degree of freedom at work, and social support) of the Copenhagen Psychosocial Questionnaire, each made up of a certain combination of questions, were used in the study. Each question had five possible response options (*always*, *often*, *sometimes*, *rarely*, *never* or *correct*, *almost correct*, *somewhat correct*, *almost wrong*, *wrong*). Answers were transformed into a number between 0 and 100. An overall scale score was computed as the mean score across questions in each scale. Depending on the mean scale scores, the respondents were divided into three groups based on the margins of tertiles: high, average, and low levels of observed phenomena (Fig. 1). The internal reliability of all five scales and the Nordic Musculoskeletal Questionnaire scale was good (Cronbach’s α > 0.7).

### Instrument of ergonomic investigation

During this study, the Rapid Upper Limb Assessment (RULA) [30] was used in order to evaluate the work posture and performed movements among computer workers. If the investigator was not able to determine it during surveillance, the employee was asked a few questions, e.g., how much time a day an employee spends talking on the phone pinching it to the ear with the help of the shoulder, while performing the routine tasks with the computer; how long he/she daily spends arranging the documents or talking to clients etc. without using a computer. Following this method, the work posture and movements of individual areas of the body were evaluated in scores with a higher score indicating greater stress for the MS area under investigation.

### Statistical analysis

Statistical data analysis was performed using the SPSS (version 20.0) software package. Hypotheses about the equality between the averages of two quantitative variables and two percentage variables were examined by U (non-parametric Mann–Whitney test) and z tests, respectively. Values of *P* < 0.05 were considered statistically significant. In order to determine whether the selected individual and work-related factors were associated with MS complaints, binary logistic regression analysis (multivariate) was applied. The results of the analysis are presented as odds ratios (ORs) and 95 % confidence intervals (CIs). The goodness-of-fit of the binary logistic regression models was evaluated using the Hosmer-Lemeshow test.

## Results

**Fig. 2:**
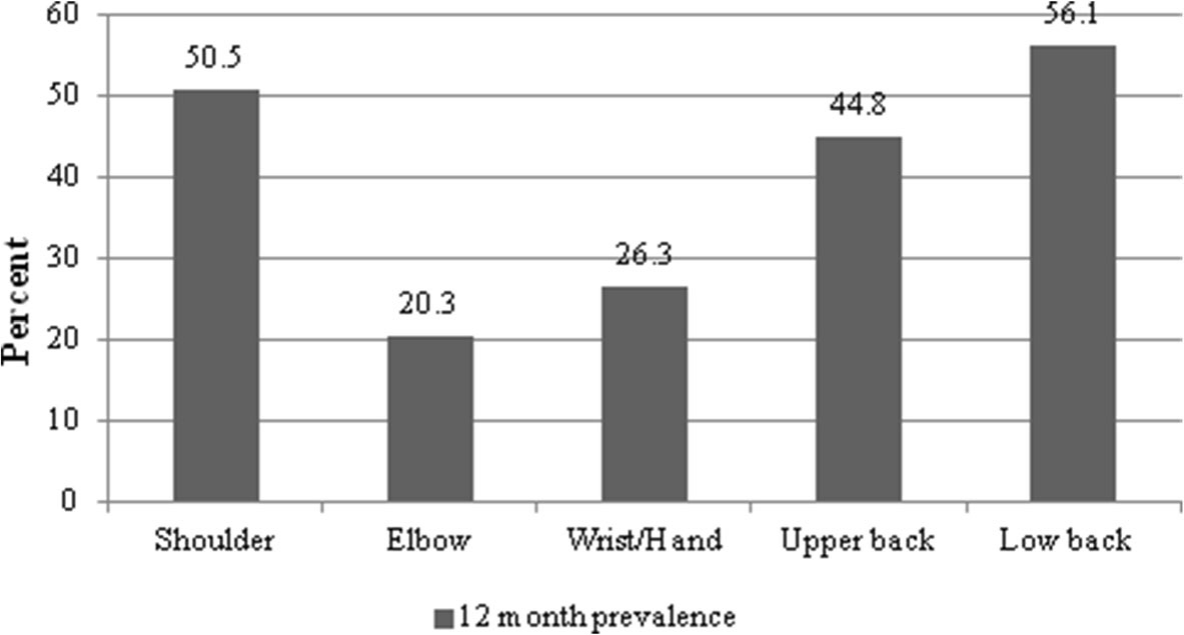
Prevalence of musculoskeletal pain by different anatomical regions

The overwhelming majority of the study population was women (94.7 %) with a mean age of 45.9 ± 11.1 years and mean computer work experience of 10.7 ± 5.5 years (Table 1). The majority of the respondents estimated they worked with a computer more than 6 h per day and did not have a brake every 2 working hours. The distribution analysis showed that about quarter of employees reported weak social support, one-third, high job demands and nearly half, low degree of freedom at work (Table 1). More than half of the employees complained about shoulder and low back pain, while elbow pain was least prevalent with one-fifth of the respondents complaining about it (Fig. 2).

**Table 1:** Individual and work-related characteristics of the study population

Factors	N (%)
Individual	
Gender	
Men	27 (5.3)
Women	486 (94.7)
Age	
23-29 years	55 (10.7)
30-39 years	91 (17.7)
40-49 years	149 (29.1)
50-70 years	218 (42.5)
Computer work experience	
1-5 years	115 (22.4)
6-15 years	287 (55.9)
16-36 years	111 (21.7)
BMI	
< 18.5 kg/m^2^	19 (3.7)
18.6-24.9 kg/m^2^	255 (49.7)
> 25 kg/m^2^	239 (46.6)
Work-related	
Duration of working with a computer	
< 4 h/day	20 (3.9)
4-6 h/day	88 (17.2)
> 6 h/day	405 (78.9)
Taking a break every 2 h	
Yes	69 (13.5)
No	444 (86.5)
Quantitative demands	
Low	208 (40.5)
Average	143 (27.9)
High	162 (31.6)
Cognitive demands	
Low	141 (27.5)
Average	191 (37.2)
High	181 (35.3)
Responsibility demands	
Low	142 (27.7)
Average	170 (33.1)
High	201 (39.2)
Degree of freedom at work	
Low	242 (47.2)
Average	157 (30.6)
High	114 (22.2)
Social support	
Weak	138 (26.9)
Average	183 (35.7)
Strong	192 (37.4)

The prevalence of MS symptoms was analyzed taking into account individual and work-related factors (Table 2). The youngest respondents significantly less frequently complained about pain in the shoulder than their older counterparts. Elbow pain was documented more frequently among employees working with a computer for more than 5 years. A BMI greater than 25 kg/m^2^ had significant associations only with low back pain. Duration of working with a computer was significantly associated only with the prevalence of MS pain in the shoulder: the employees working with a computer more than 4 h per day more frequently complained about pain in this area. Wrist/hand and low back complains were more common among those who did not have any break every 2 h. The respondents who reported low quantitative demands had pain less frequently in all investigated anatomical areas except for the low back. Low cognitive and responsibility demands were associated with less frequent pain in the shoulder, elbow, and upper back areas. Significantly higher frequencies of complaints in the shoulder and wrist/hand areas were observed for respondents reporting a low degree of freedom at work. Respondents with weak social support had a higher frequency of MS complaints in the low back area (Table 2).

**Table 2:** Prevalence of musculoskeletal pain with respect to individual and work-related factors

Factors	Shoulder N (%)	Elbow N (%)	Wrist/hand N (%)	Upper back N (%)	Low back N (%)
Individual					
Gender					
Men	4 (14.8)	2 (7.4)	4 (14.8)	9 (45.5)	15 (55.6)
Women	255 (52.5)*	102 (21.0)	131 (27.0)	221 (33.3)	273 (56.2)
Age					
23-29 years	19 (34.5)	5 (7.3)	12 (21.8)	19 (34.5)	30 (54.5)
30-39 years	42 (46.2)*	10 (11.0)	21 (23.1)	38 (41.8)	48 (52.7)
40-49 years	75 (50.3)*	36 (24.2)*	46 (28.9)	84 (56.4)*	86 (57.7)
50-70 years	123 (56.4)*	54 (24.8)*	59 (27.1)	89 (40.8)	124 (56.1)
Computer work experience					
1-5 years	48 (41.7)	14 (1.2)	26 (22.6)	40 (34.8)	62 (53.9)
6-15 years	152 (53.0)	65 (22.6)*	80 (27.9)	144 (50.2)*	165 (57.5)
16-36 years	59 (53.2)	25 (22.5)*	29 (26.1)	46 (41.4)	61 (55.0)
BMI					
< 18.5 kg/m^2^	9 (47.4)	0 (0)	4 (21.1)	6 (31.6)	8 (42.1)
18.6-24.9 kg/m^2^	128 (50.2)	50 (19.6)	71 (27.8)	122 (47.8)	133 (51.0)
> 25 kg/m^2^	122 (51.0)	54 (22.6)	60 (25.1)	102 (41.7)	144 (60.3)*
Work-related					
Duration of working with a computer					
< 4 h/day	6 (30)	6 (30.0)	5 (25.0)	8 (40)	8 (40.0)
4-6 h/day	47 (53.4)*	14 (15.9)	17 (19.3)	36 (40.9)	55 (62.5)
> 6 h/day	206 (50.9)*	84 (20.7)	113 (27.9)	186 (45.9)	225 (55.6)
Taking a break every 2 h					
Yes	30 (43.5)	15 (21.7)	13 (18.8)	26 (37.7)	31 (44.9)
No	229 (51.6)	89 (20.0)	122 (27.5)*	204 (45.9)	257 (57.9)*
Quantitative demands					
Low	84 (40.4)	32 (15.4)	42 (20.2)	73 (35.1)	112 (53.8)
Average	80 (55.9)*	30 (21.0)	37 (25.9)	69 (48.3)*	79 (55.2)
High	95 (58.6)*	42 (25.9)*	56 (34.6)*	88 (54.3)*	97 (59.9)
Cognitive demands					
Low	59 (41.8)	17 (12.1)	31 (22.0)	56 (39.7)	74 (52.5)
Average	96 (50.3)	41 (21.5)*	54 (28.3)	78 (40.8)	114 (59.7)
High	104 (50.5)*	46 (25.4)*	50 (27.6)	96 (53.0)*	100 (55.2)
Responsibility demands					
Low	63 (44.4)	23 (16.2)	35 (24.6)	51 (35.9)	80 (56.3)
Average	79 (46.5)	28 (16.5)	45 (26.5)	81 (47.6)*	98 (57.6)
High	117 (58.2)*	53 (26.4)*	55 (27.4)	98 (48.8)*	110 (54.7)
Degree of freedom at work					
Low	135 (55.8)*	52 (21.5)	72 (29.8)*	114 (47.1)	138 (57.0)
Average	73 (46.5)	33 (21.0)	40 (25.5)	69 (43.9)	87 (55.4)
High	51 (44.7)	19 (16.7)	23 (20.2)	47 (41.2)	63 (55.3)
Social support					
Weak	75 (54.3)	31 (22.5)	37 (26.8)	73 (52.9)*	89 (64.5)*
Average	85 (46.4)	34 (18.6)	46 (25.1)	77 (42.1)	101 (55.2)
Strong	99 (51.6)	39 (20.3)	52 (27.1)	80 (41.7)	98 (51.0)

z test, *P < 0.05 - comparing with: youngest age group; smallest work experience group; 18.6-24.9 kg/m^2^ BMI group; <4 h/day working with computer respondents group; those who taking break every two hours; working in a positive work environment with respect to current psychosocial factor

**Fig. 3:**
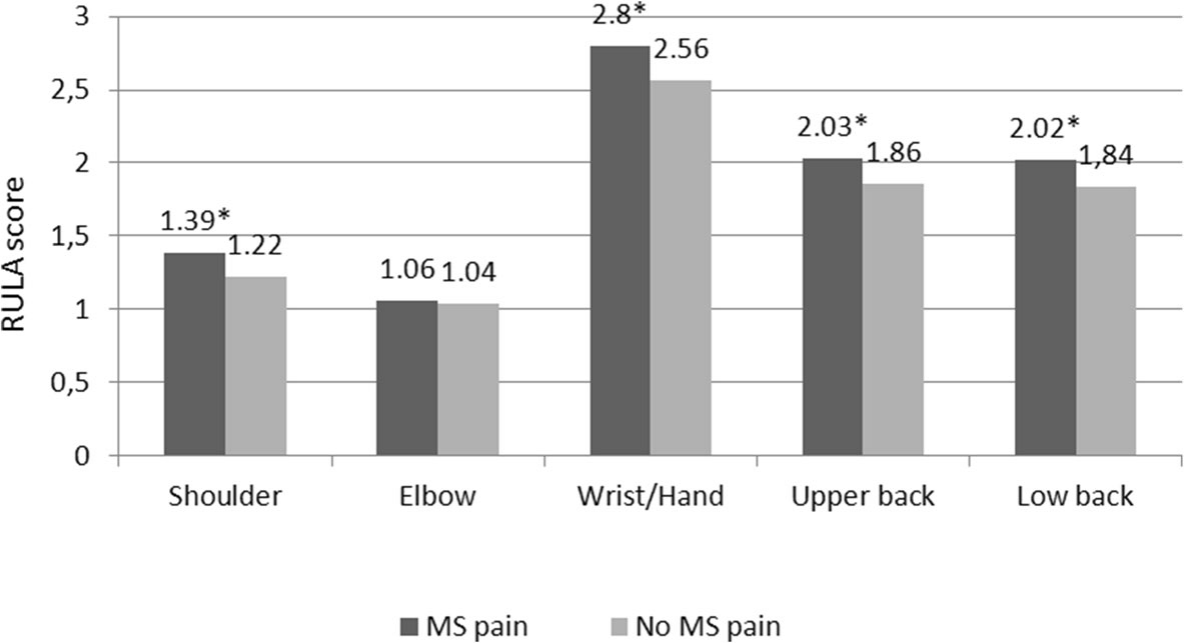
Mean RULA posture and movement scores in the groups of respondents with and without musculoskeletal (MS) pain

The RULA score significantly differed comparing the groups of respondents with and without MS pain (Fig. 3). The respondents experiencing pain in shoulder, wrist/hand, upper back, and low back areas had a statistically significantly higher mean RULA score.

Table 3 shows the results of logistic regression analysis investigating associations between individual and work-related factors, and pain in different anatomical areas. The results of Hosmer-Lemeshow test revealed good fits for all models (*χ* ^2^ = 7.70, *df* = 8, *P* = 0.46 for shoulder model; *χ* ^2^ = 10.02, *df* = 8, *P* = 0.26 for elbow model; *χ* ^2^ = 4.61, *df* = 8, *P* = 0.79 for wrists/hand model; *χ* ^2^ = 3.16, *df* = 8, *P* = 0.92 for upper back model; *χ* ^2^ = 5.78, *df* = 8, *P* = 0.67 for low back model).

**Table 3:** Logistic regression model for musculoskeletal pain in various anatomical regions (fully adjusted)

Factor	Musculoskeletal region									
	Shoulder		Elbow		Wrist/hand		Upper back		Low back	
	OR	95 % CI	OR	95 % CI	OR	95 % CI	OR	95 % CI	OR	95 % CI
Gender										
Men	1		1		1		1		1	
Women	**6.1**	**1.95-19.08**	2.95	0.59-14.58	1.52	0.491-4.88	1.21	0.50-2.93	1.12	0.48-2.61
Age										
23-29 years	1		1		1		1		1	
30-39 years	1.48	0.65-3.37	1.15	0.30-4.34	0.78	0.30-2.05	0.80	0.34-1.86	0.92	0.41-2.06
40-49 years	1.89	0.65-3.53	2.94	0.62-8.21	1.09	0.41-2.91	1.62	0.68-3.85	1.07	0.46-2.46
50-70 years	**2.16**	**1.02-4.99**	**3.38**	**1.87-12.05**	1.11	0.41-2.00	0.93	0.39-2.23	0.91	0.42-2.23
Computer work experience										
1-5 years	1		1		1		1		1	
6-15 years	1.03	0.56-1.86	1.49	0.66-3.37	1.31	0.65-2.62	**1.87**	**1.01-3.46**	0.90	0.50-1.63
16-36 years	1.04	0.51-2.09	1.46	0.58-3.62	1.16	0.52-2.60	1.42	0.70-2.91	0.81	0.41-1.61
BMI										
< 18.5 kg/m^2^	0.79	0.50-1.25	0.88	0.53-1.46	0.77	0.22-2.61	0.75	0.24-2.31	1.48	0.55-3.99
18.6-24.9 kg/m^2^	1		1		1		1	1	1	
> 25 kg/m^2^	1.44	0.75-2.77	1.02	0.64-2.45	0.17	0.45-1.15	0.70	0.46-1.08	**1.50**	**1.24-4.28**
Duration of working with a computer										
< 4 h/day	1		1		1		1		1	
4-6 h/day	**3.03**	**1.97-9.40**	0.41	0.14-1.70	0.46	0.17-1.90	0.70	0.23-2.13	2.14	0.76-6.01
> 6 h/day	2.40	0.81-7.11	0.59	0.22-2.13	0.70	0.22-2.22	0.93	0.32-2.73	1.58	0.59-4.21
Taking a break every 2 h										
Yes	1		1		1		1		1	
No	0.80	0.44-1.46	0.61	0.29-1.27	1.31	0.63-2.68	0.95	0.51-1.76	1.65	1.16-2.95
RULA score	**1.68**	**1.20-2.36**	1.62	0.69-3.81	**1.59**	**1.15-2.21**	**1.38**	**1.04-1.75**	**1.30**	**1.03-1.65**
Quantitative demands										
Low	1		1		1		1		1	
Average	**1.67**	**1.05-2.68**	1.27	0.70-2.95	1.2	0.70-2.07	**1.68**	**1.04-2.71**	0.88	0.55-1.41
High	**1.83**	**1.14-2.92**	**1.75**	**1.12-3.11**	**1.86**	**1.11-3.12**	**2.00**	**1.25-3.20**	1.09	0.69-1.74
Cognitive demands										
Low	1		1		1		1		1	
Average	1.17	0.73-1.89	1.52	0.78-2.95	1.08	0.62-1.86	0.75	0.46-1.22	1.22	0.76-1.96
High	1.20	0.71-2.04	1.51	0.76-3.00	0.86	0.47-1.56	1.20	0.71-2.04	1.01	0.60-1.68
Responsibility demands										
Low	1		1		1		1		1	
Average	1.18	0.72-1.93	1.164	0.60-2.22	1.24	0.72-2.14	**1.78**	**1.08-2.92**	1.11	0.69-1.79
High	**1.89**	**1.14-3.14**	**2.04**	**1.09-3.82**	1.21	0.69-2.11	**1.54**	**1.06-2.57**	1.09	0.66-1.78
Degree of freedom at work										
Low	0.94	0.55-1.59	1.61	0.78-2.82	1.25	0.66-2.34	1.26	0.71-2.23	1.05	0.64-1.71
Average	1.39	0.84-1.59	1.48	0.81-3.19	1.44	0.81-2.56	1.34	0.73-2.21	1.05	0.62-1.76
High	1		1		1		1		1	
Social support										
Weak	1.35	0.82-2.16	1.31	0.62-1.93	0.88	0.53-1.46	**1.74**	**1.07-2.83**	**1.86**	**1.15-3.00**
Average	0.97	0.61-1.52	1.10	0.73-2.35	0.66	0.56-1.63	1.10	0.70-1.75	1.22	0.79-1.90
Strong	1		1		1		1		1	

Women and 50–70-year olds were 6 and 2 times more likely than men and older respondents, respectively, to experience MS pain in the shoulder. Moreover, time spent working with a computer 4–6 h a day, higher levels of quantitative demands, and high level of responsibility demands were associated with an increased risk of having shoulder pain. For each one-point increase in the RULA score, the likelihood of having shoulder pain increased by 68 %.

Being a 50–70-year old and high levels of quantitative and responsibility demands were associated with a greater risk of experiencing elbow pain.

The likelihood of having wrist/hand pain was positively associated with a high level of quantitative demands. For each one-point increase in the RULA score, the likelihood of having wrist/hand pain increased by 59 %.

Computer work experience of 6–15 years, higher levels of quantitative and responsibility demands, and weak social support at work were found to be associated with a greater odds of experiencing upper back pain. For each one-point increase in the RULA score, the likelihood of having upper back pain increased by 38 %.

Having a BMI of >25 kg/m^2^, no taking a break every 2 h, and weak social support at work were associated with an increased risk of having low back pain. For each one-point increase in the RULA score, the likelihood of having low back pain increased by 30 %.

## Discussion

The aim of this cross-sectional study was to evaluate the prevalence of MS pain in various anatomical areas among computer workers in the public sector of Kaunas County. This study showed a high prevalence of MS pain in body parts such as shoulders, upper back, and low back among office workers. According to numerous studies, neck pain is in the leading position with the prevalence ranging from 19 % to 70 % in the population of office workers [4, 5, 8, 11, 12, 21, 31–36]. The prevalence of shoulder pain in this study was also high (50 %), and this is in line with the finding of Australian [4] and Chinese [31] studies, while Finnish [5] and German [37] studies documented a lower prevalence of MS complaints in this area. It is worth noting that some epidemiological studies report the prevalence of neck and shoulder complaints or complaints in the arm, neck, and shoulder areas together because of similar etiology factors. In different studies, the prevalence of pain in these localizations was also about 50 % [10, 33, 38]. Pain in arm, wrist, and hand areas is also common, and typically about 30 % of computer workers have MS complaints in these anatomical regions [4, 31, 33]. In our study, about quarter of the respondents complained about pain in elbow and hand/wrist regions (20.6 % and 26.3 %, respectively). Back pain is a very common MS complaint in the general population as well, and according to a systematic review by Walker, which included 53 studies, it ranges between 22 % and 65 % [39], showing that there are a lot of predisposing risk factors associated not only with the work environment, but also with domestic or other activities [20]. Our research also showed a high prevalence rate of back pain accounting for 44.8 % in the upper back and 56.1 % in the low back.

Since computerization levels in the office work environment have dramatically increased, the question of a multifactorial origin of MS disorders is being discussed by scientists. There is evidence that all predisposing factors – individual, ergonomic, and psychosocial – are related to the development of MS complaints [3, 23, 24]. The results of our study also confirmed that MS pain was associated with individual and ergonomic as well as psychosocial factors among computer workers.

Contrary to previous studies that confirmed differences between genders and documented that women are more likely to experience MS pain [4–6, 8, 20–22, 40], our study showed that only shoulder pain was more frequent among women and this was confirmed by the results of multivariate logistic regression as well. However, it should be mentioned that women comprise a major proportion of Lithuanian public office workers, and in our study, men accounted only for 5.3 % of the overall study population. Because of this limitation, the distribution of MS pain in the male group could not reflect a real situation.

In our study, the frequency of complaints in almost all anatomical regions was higher in older and oldest respondents’ age groups as compared with the youngest group. Moreover, multivariate logistic regression analysis showed that the oldest participants (50–70 years old) were more than 2 and 3 times as likely to have MS complaints in shoulder and elbow areas. Some epidemiological studies have reported that middle-aged employees are most vulnerable to pain in neck and shoulder localizations [4, 8, 41–43]. One could assume that the respondents of this particular age group have the largest experience of working with computers; however, our study did not confirm that biggest work experience associated with higher prevalence of MS pain.

Among individual risk factors, also BMI and its relationship with MS pain were investigated. A BMI of >25 kg/m^2^ was found to be associated with MS pain in the low back anatomical region, and this is in agreement with other epidemiological studies [20, 44].

During comprehensive assessment of the risk of complaints about MS pain, it is important to take into account ergonomics at the workplace for those who work with a computer. Objective investigations on muscular activity have documented increased muscle tension during computer work [40, 45–47]. Other epidemiological studies have shown that improper localization of equipment in the computerized workplace is associated with MS pain [48, 49].

Due to the fact that not only inadequacy of the workstation can determine the worker’s posture and movements, we assessed the ergonomics of computer work by the RULA method, which evaluates harmful posture and movements for certain areas of the body. The main strength of this study was ergonomic evaluation with the RULA method applied by the investigators in order to achieve greater objectivity. The results showed that a higher RULA score was statistically significantly associated with a greater risk of having MS complaints in the shoulder, wrist/hand, upper back, and low back anatomical areas.

In many epidemiological studies, MS pain in shoulder area was investigated together with neck complaints; therefore, work posture and movements were identified as significant factors having an impact on pain in both neck and shoulder areas [13, 14]. In our study, the duration of work with a computer for 4–6 h a day increased the likelihood of experiencing pain in the shoulder area, but Blatter et al. showed that employees who work with a computer more than 6 h per day were at increased risk of shoulder pain [50]. Awkward posture and movements of computer workers have been confirmed as significant risk factors for MS pain in the arm area [10, 15, 32, 51, 52]; however, our data showed significant associations these factors and pain only in hand/wrist anatomical area.

The neck and shoulders are the most affected anatomical regions of the human body in computer workers, and for this reason, MS pain in the back including both upper and low back is less investigated in scientific studies involving computer workers as a study population. Despite this, some epidemiological studies [4, 32, 49] as well as our data confirmed associations between MS pain in the back area and inadequate work posture and movements.

Development of information technologies and computerization has led to many changes in office workers’ professional practice and constantly increasing job demands. Negative consequences of computerization due to increasing workload, employer expectations, or job tension to employees were recognized [53], and high job demands were found to be associated with MS symptoms in many populations of office workers [16, 10, 54]. Our study also demonstrated that quantitative job demands were significantly associated with MS complaints in almost all investigated anatomical areas except for the low back. Responsibility demands were found to be a significant factor for shoulder, elbow, and upper back complaints, while weak social support had a significant impact on both upper and low back pain. The observation that the odds ratios of all MS pain-predisposing factors – individual, psychosocial, and ergonomic – were almost of equal magnitude suggest that their contribution to etiology of MS pain is very similar.

## Conclusions

The prevalence of MS pain among computer users was high, with shoulders and low back being the most affected anatomical areas. Significant associations between individual factors, work ergonomics (inappropriate posture and movements), and MS pain were found. Work-related psychosocial factors had a significant impact on experiencing pain as well: high quantitative demands were associated with MS complaints in almost all anatomical areas, and weak social support was a significant predictor for MS complaints in the upper and low back areas.

Preventive measures at the workplace should be directed to the improvement in ergonomic work environment and reducing job strain caused inadequate workload, high responsibilities, and weak social support.

## Abbreviations

BMI: Body mass index
CI: Confidence interval
MS: Musculoskeletal
OR: Odds ratio
RULA: Rapid upper limb assessment
